# Train to sustain: a randomised controlled trial evaluation of a vitality training employing behaviour-change techniques

**DOI:** 10.3389/fpsyg.2023.1320826

**Published:** 2024-01-16

**Authors:** Bernice R. C. Plant, Mattheis L. Van Leeuwen, Pascale Peters, Beatrice I. J. M. Van der Heijden

**Affiliations:** ^1^BehaviourWorks Australia, Monash Sustainable Development Institute, Monash University, Clayton, VIC, Australia; ^2^Behavioural Science Institute, Radboud University, Nijmegen, Netherlands; ^3^Nyenrode Business Universiteit, Breukelen, Netherlands; ^4^Department of Organisation, Leadership and Management, Inland School of Business and Social Sciences, Lillehammer, Norway; ^5^Institute for Management Research, Radboud University, Nijmegen, Netherlands; ^6^Faculty of Management, Open Universiteit, Heerlen, Netherlands; ^7^Department of Marketing, Innovation and Organisation, Ghent University, Ghent, Belgium; ^8^Business School, Hubei University, Wuhan, China; ^9^Kingston Business School, Kingston University, Kingston upon Thames, United Kingdom

**Keywords:** behaviour change, burnout, randomised controlled trial, self-efficacy, vitality, work-related stress

## Abstract

**Introduction:**

This paper reports on the effects of a 9-week vitality training that employed behaviour-change techniques and was evaluated using a randomised controlled trial (RCT) in three large companies based in the Netherlands.

**Methods:**

A total of 84 adult employees from three participating organisations in the Netherlands were enrolled in the study. A parallel group RCT design was employed and participants were assigned using individual random assignment to either an intervention (*n* = 38) or a waitlist control group (*n* = 46). The intervention consisted of a 9-week vitality training employing the behaviour-change techniques of self-persuasion, implementation intentions, and self-efficacy, which was delivered in-house over five fortnightly 2-hour sessions. Primary outcomes (i.e., reported energy and stress) and secondary outcomes (i.e., reported daily life satisfaction and work capacity) were assessed prior to, immediately after, and 3 months following the intervention.

**Results:**

A mixed MANOVA revealed a significant interaction effect between treatment group and time period for the combination of reported energy, stress, daily life satisfaction, and work capacity. Subsequent univariate ANOVAs revealed significant interactions between treatment group and time period for reported energy, stress, and daily life satisfaction; however, not for reported work capacity. Improvements in outcomes were observed for both groups following their completion of the vitality training; however, not all improvements reached statistical significance. Reported self-efficacy regarding managing work-life balance was found to mediate the relationship between the effects of the intervention and reported energy; however, such an effect was not found for stress.

**Discussion:**

An intervention drawing upon evidence-based behaviour-change techniques shows promise for improving indicators associated with burnout; although, it is recommended that in future research a larger-scale evaluation be conducted over a longer time period with an active control group to establish effectiveness.

**Clinical trial registration:** https://www.anzctr.org.au/, ACTRN12622001268730.

## 1 Introduction

Vitality has important implications for individuals’ personal lives as well as for their working lives and is positively related to one’s physical and mental health (Ryan and Frederick, 1997), wellbeing, and life satisfaction (as per Guérin’s model, see Guérin, 2012). In the workplace, vitality is related to thriving (Kleine et al., 2019), work engagement (see the Job Demands-Resources model, Bakker et al., 2014), and reduced burnout levels (Demerouti et al., 2001; Kristensen et al., 2005). Consequently, enhancing employee vitality not only benefits individuals but also the organisations for which they work.

The Job Demands-Resources (JD-R) theory is a unifying job design theory that explains how job demands and resources can influence job performance through employee wellbeing, and how employees use proactive as well as reactive work behaviours to influence the job demands and resources they face (Bakker et al., 2014; Bakker and Demerouti, 2017). The JD-R theory originated from the engagement and burnout literatures (Demerouti et al., 2001), and, over the past two decades, JD-R theory “has been able to synthesize knowledge from various theories of job stress and work motivation, including two-factor theory (Herzberg, 1966), job characteristics theory (Hackman and Oldham, 1976), the job demands–control model (Karasek, 1979), the effort–reward imbalance model (Siegrist, 1996), and conservation of resources theory (Hobfoll et al., 2018). Bakker et al. (2023, p. 32) as a theoretical framework that helps to synthesise previous theorising on job stress and motivation, the JD-R theory provides a comprehensive understanding and has been used as the explanatory lens in our scholarly work.

We build on the notion that vitality, thriving, work engagement, and reduced burnout levels are, in turn, related to important work outcomes, including job performance (Taris, 2006; Christian et al., 2011) and turnover intentions (Wefald et al., 2012). In particular, we address a gap in the literature by investigating whether enhancing personal resources can contribute to personal wellbeing and whether this can be trained.

Although the scientific study of vitality is fairly recent (i.e., emerging in the early 1990s; Gould, 1991), vitality has been defined and measured in diverse ways in the literature. Vitality is often used interchangeably with terms such as vigour and energy, and described as either a feeling, state, or an experience (Lavrusheva, 2020). A recent scoping review of the vitality-related research domain conceptualised vitality as having the following fundamental characteristics: (i) it is subjective in nature; (ii) it is a positive experience; (iii) it fluctuates and can be restored; (iv) it can be managed or harnessed by an individual; and (v) it is simultaneously comprised of physiological and psychological energy (Lavrusheva, 2020). Given that vitality is a subjective experience, an individual’s vitality is typically assessed using self-report measures, with various scales applied across disciplines (see Lavrusheva, 2020). Examples hereof are the Subjective Vitality Scale (SVS; Ryan and Frederick, 1997), the vitality subscale of the RAND 36-Item Health Survey (SF–36; see Tarlov et al., 1989), and the vigour subscale of the Utrecht Work Engagement Scale (UWES; Schaufeli and Bakker, 2003). Despite variations in how vitality is defined and measured, energy is a core component across different contexts and scales, ranging from individual wellbeing to workplace engagement.

Numerous factors have been reported as precursors to the experience of vitality, and these have recently been organised into three overarching categories: physiological, psychological, and environmental (Lavrusheva, 2020). In the case of physiological antecedents, developing healthy lifestyle habits, such as increased sleep, exercise, fruit, and vegetable intake, has been associated with increased vitality (Strijk et al., 2009; Smolders et al., 2013; Conner et al., 2017). Psychological factors associated with increased vitality include self-regulation, working from one’s own goals, and the practice of mindfulness (Fritz et al., 2011; Niessen et al., 2012). Furthermore, environmental factors associated with increased vitality include aspects of the work context, such as meaningful work and learning something new, as well as leisure activities (activities in the natural environment and during weekends) (Sheldon et al., 1996; Ryan et al., 2010). These findings have important implications for designing interventions to increase vitality.

For instance, that work situations can drain or replenish an individual’s vitality highlights the importance of building individuals’ capabilities to recognise and manage situations that affect their energy levels (Op den Kamp et al., 2018). Furthermore, the diversity of factors highlights the necessity of adopting holistic and personalised approaches to address multiple factors and tailor interventions to meet diverse individual needs. Importantly, many of the aforementioned precursors to vitality may be positively influenced by individuals performing specific actions (*cf.* internal circumstances that may be more difficult to change, such as personality traits, see Ryan and Frederick, 1997; Tasselli et al., 2018). This suggests that person-directed interventions could benefit from employing behaviour-change strategies (Abraham and Michie, 2008) to enable individuals to self-manage situations in their work or non-work lives to enhance their vitality.

The proposed behaviour-change approach draws parallels with interventions targeting ‘job crafting’ behaviours (Tims and Bakker, 2010; Van den Heuvel et al., 2015; Devotto and Wechsler, 2019), where individuals actively shape their work tasks or relationships, and/or employ cognitive crafting (Zhang and Parker, 2019) to improve work engagement. A recent meta-analysis by Oprea et al. (2019) provides strong evidence for the relationship between job crafting interventions and their components with work engagement and job performance. Despite this, insights from a systematic review (Devotto and Wechsler, 2019) enrich our understanding about the potential moderating role of *intervention focus* on outcomes. As an example: Interventions that focussed on gaining job and personal resources and cognitions, such as increased meaning, were associated with enhanced work engagement; conversely, those targeting the reduction of hindering job demands did not have a similar effect on work engagement but had a positive effect on health outcomes. These variations emphasise the importance of tailoring crafting behaviours to individual needs, considering specific motivational (e.g., work engagement) or health-related requirements.

Interestingly, evidence from the work-life balance literature suggests that improvements in work life may spill over into non-work life and vice versa (Sirgy and Lee, 2018). Applied to the domain of vitality, this suggests that it may not be necessary for individuals to focus *exclusively* on managing situations in the workplace to experience benefits in their working life. In view of this, a vitality training employing behaviour-change techniques could take a whole-life perspective (Hirschi et al., 2020) and consider the intersection of work and non-work roles (Greenhaus and Kossek, 2014), which would allow employees to tailor the focus of the vitality training to their specific needs—whether their vitality needs fell in the area of work or in their non-work life. This is important in light of protecting and further enhancing one’s career sustainability across the lifespan (De Vos et al., 2020; Van der Heijden et al., 2020).

A number of reviews (Maricuţoiu et al., 2016; Dreison et al., 2018) have called for controlled evaluations of interventions, such as randomised controlled trials (RCTs), and for the inclusion of follow-up measures to examine their longer-term effects (e.g., 1–6 months after intervention completion). Particularly in the burnout literature, experts have advocated for the development, implementation, and evaluation of a broader range of interventions (Dreison et al., 2018), particularly tailored to individual participants (Maricuţoiu et al., 2016) to address both individual and organisational needs (Dreison et al., 2018). That is, although a ‘one-size-fits-all’ intervention approach may be desirable for the internal validity of an evaluation, an intervention’s effects may be diminished if they are not carefully aligned to the needs of the participants. To this end, a vitality training that applies an evidence-based method, while allowing participants to target their individual needs in using these methods, may be valuable. To the best of our knowledge, no previous interventions to enhance vitality have employed behaviour-change techniques that would enable such an approach.

Our study aims to explore the effects of a novel vitality training that incorporates behaviour-change techniques on employees’ reported energy and subjective experiences of stress, daily life satisfaction, and work capacity. In doing so, we contribute to the scholarly literature in the field of JD-R, specifically by advancing empirical knowledge on the value of proactive vitality management. While previous research has focused on determining the role of job demands and job resources in explaining employee wellbeing, and consequently, job performance, proactive vitality management focuses on the role of changing the employees themselves. Whereas job crafting (Tims and Bakker, 2010; Van den Heuvel et al., 2015; Devotto and Wechsler, 2019) is aimed at changing the situation in terms of job demands and job resources, proactive vitality management, as proposed in recent years by JD-R theory (Bakker et al., 2023), is mainly aimed at *improving employees’ personal physical and psychological resources* to promote optimal functioning at work (*ibid.*). As such, we build on JD-R theory to explain employee wellbeing via the enhancement of personal resources.

In addition, we sought to address methodological limitations of previous evaluations by employing an RCT design and by exploring whether any observed effects of the intervention were maintained 3 months following its implementation. To estimate the immediate and longer-term effects of the intervention, we employed sequential training phases for the intervention and waitlist control groups, and assessed outcomes before the intervention, immediately after, and 3 months following it. As the training was designed to improve participants’ vitality, reported energy and stress were considered as primary outcome variables for this study. Given that vitality is expected to influence participants’ wellbeing and work functioning over time, daily life satisfaction, and work capacity were included as secondary outcomes to explore the broader impacts of our training. We hypothesised that the intervention would significantly improve the aforementioned outcome measures as follows:

*Hypothesis 1 (H1)*: A significant interaction effect will be observed between treatment group and measurement time period for the combination of reported energy, stress, daily life satisfaction, and work capacity.

*Hypothesis 2 (H2)*: The intervention will have a significant effect on our primary outcome measures of reported energy (*H2*a) and stress (*H2*b), resulting in a significant increase in reported energy and a significant decrease in reported stress for the intervention group (Time Point 0 to Time Point 1), and later for the control group (Time Point 1 to Time Point 2).

*Hypothesis 3 (H3)*: The intervention will have a significant effect on our secondary outcome measures of daily life satisfaction (*H3*a) and work capacity (*H3*b), resulting in significant increases in reported daily life satisfaction and work capacity of the intervention group (Time Point 0 to Time Point 1), and later for the control group (Time Point 1 to Time Point 2).

From a behaviour-change perspective, training-based interventions serve a function of building capability (Michie et al., 2011), and skill enhancement and application contributes to the development of self-efficacy (Bandura, 1990; Bandura and Locke, 2003). Through this lens, improvements in reported energy and stress levels following training in proactive vitality management may be linked to an increased belief in one’s ability to proactively manage and balance their own work-life situation. As such, we explored whether self-efficacy to manage one’s work-life balance mediated the effects of the intervention on our primary outcome measures of energy and stress. Finally, we explored the acceptability of the intervention by examining participants’ subjective experiences of the vitality training method and its effects.

Therefore, our study contributes to the scholarly literature in this field in the following ways. First, we use a novel training method comprised of evidence-based behaviour-change techniques (Abraham and Michie, 2008). Second, we use a training method that allows individuals to tailor the focus of the training to their individual needs (Taylor and O’Driscoll, 1998). Third, we explore the effects of the training using an RCT, including assessments 3 months following the intervention. Fourth, we explore whether reported self-efficacy is a mechanism by which the training influences reported energy and stress (*cf.* Strecher et al., 1986).

## 2 Materials and methods

### 2.1 Participants

Participants were employees from three large organisations based in the Netherlands, who volunteered to take part in an intervention advertised as a vitality training. The sample size was determined on a pragmatic basis, including employees across participating organisations who elected to complete the training and participate in the study. A total of 84 employees (52 females, 32 males), with a mean age of 47.39 years (age range 29–62 years) enrolled and participated in the study. The types of organisations that participants worked for included education (*n* = 21), commercial (*n* = 43), and government (*n* = 20). The educational training of the participants was largely above higher vocational level. The average number of years worked in the organisation was 13.36 years (SD = 9.84).

### 2.2 Design

**Figure 1:**
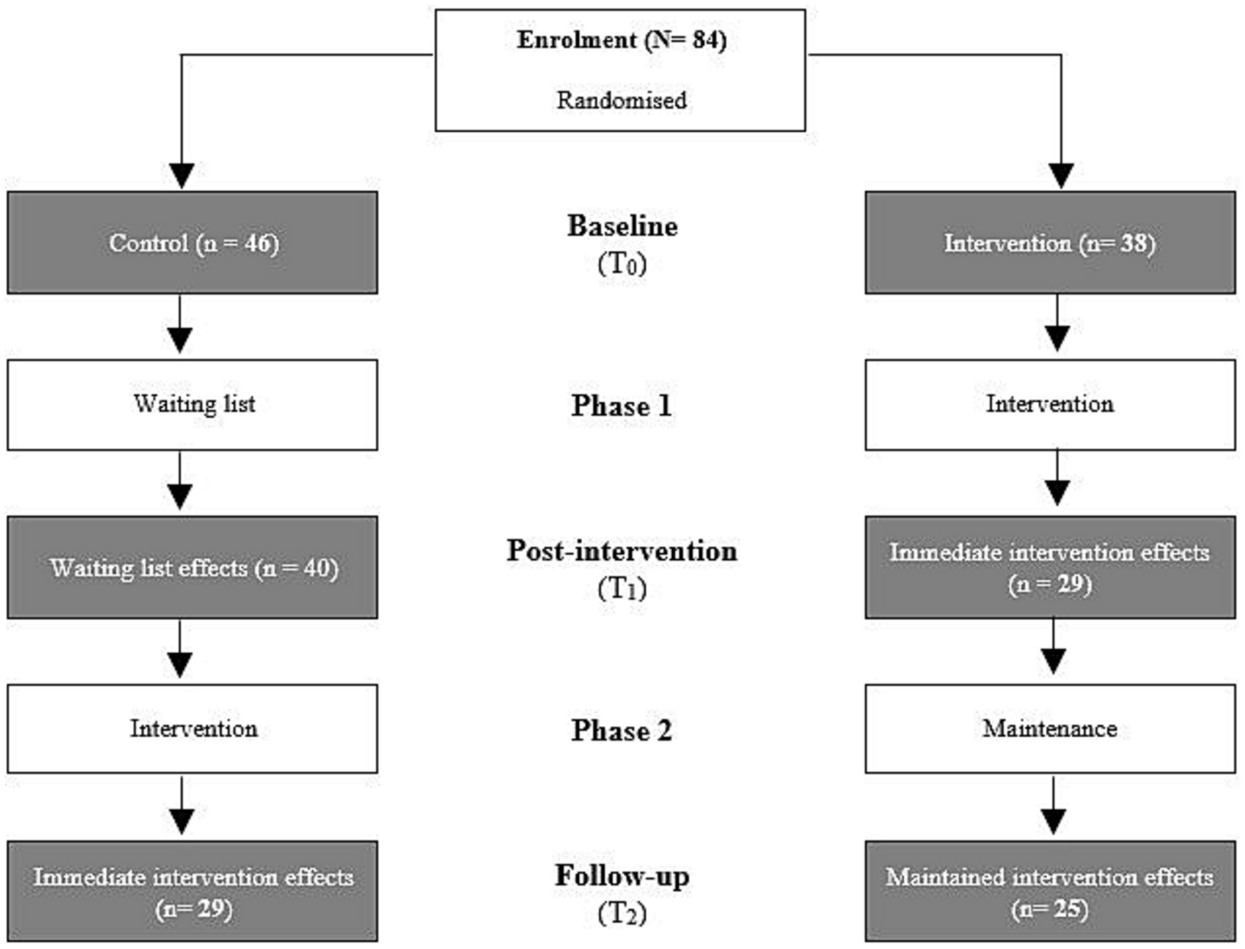
Overview of the design of the vitality training evaluation study.

A parallel group RCT design was used, with two phases, and with individual random assignment by the trainer to an intervention group (*n* = 38) or a waitlist control group (*n* = 46) within their organisation. A waitlist control group was used to control for traditional threats to internal validity, as this was ethically appropriate and reported as useful for initial evaluations of novel interventions (Mohr et al., 2009). Intervention allocation was concealed from participants, but not from data analysts. A flowchart of the study design is presented in Figure 1. The intervention group commenced the vitality training during Phase 1 and the control group was put on a waiting list for the training during this time. During Phase 2, the control group completed the vitality training and the intervention group was on a maintenance phase. The outcome measures were assessed at three time points during the trial (T_0_–T_2_ in Figure 1), each with a 2-week data collection period, allowing changes in these measures to be assessed within and between the groups before and after the intervention, as well as during a 3-month follow-up for the intervention group. All measures were assessed in Dutch and were completed anonymously using Qualtrics.^1^ The study was conducted in the Netherlands in full compliance with the applicable rules of the institutional review board (Ethics Committee Faculty of Social Sciences, Radboud University, the Netherlands) and informed consent was obtained from all participants. All ethical codes as maintained in the NIP (the Dutch Association of Psychologists), the American Psychological Association, and the British Psychological Society were followed.

### 2.3 Intervention: vitality training employing behaviour-change techniques

The training was designed to include scientifically-tested behaviour-change techniques that allowed participants to identify their own areas of concern, to set a limited number of personally relevant and meaningful goals, and to develop personalised strategies for change. In terms of ease of implementation and to promote habit formation (i.e., requiring repetition over time, see Lally et al., 2010), the intervention was comprised of subsequent short sessions, whereby participants could practice and evaluate their behaviour-change process over time. The overall aim of the intervention was to increase reported levels of energy and to reduce reported levels of stress as proxies for burnout symptoms.

#### 2.3.1 Techniques central to the training method

The vitality training employed a specially designed ‘method’, which required that participants worked through assignments employing evidence-based behaviour-change techniques, including self-persuasion, implementation intentions, and self-efficacy techniques (Hagger et al., 2020). These techniques were selected to: (i) increase commitment to the self-defined goals (self-persuasion); (ii) increase the chance that these goals will be put into action (implementation intentions); and (iii) increase the confidence participants have in themselves to execute these actions (self-efficacy). Each of these techniques is described in more detail below.

##### 2.3.1.1 Self-persuasion

This technique requires that individuals provide their own reason for working on a specific goal or for changing their behaviour. Self-persuasion thus draws on individuals’ personal motivations and has been shown to have greater effects on changing individuals’ behaviour than external forms of persuasion (Aronson, 1999). For example, having people generate their own reasons for why they should not smoke led to less smoking directly following the experiment compared to providing individuals with high quality arguments that were generated by others (Müller et al., 2009). The effects of self-persuasion are thought to occur due to higher personal relevance and involvement in the behaviour-change process and less resistance towards the message source (Aronson, 1999 for a review). Moreover, it has been reported that producing one to two self-generated arguments may have greater persuasive outcomes than ten (externally) provided arguments, even if the provided arguments are rated as being of better quality (Müller et al., 2017). This effect, however, only held when the number of self-generated arguments was low. That is, the effects of self-persuasion diminished if individuals generated many arguments for why they should or should not perform a particular response. Therefore, in the current intervention, participants had to write down one or two reasons why they should work on particular areas or perform particular actions they had defined earlier in the training.

##### 2.3.1.2 Implementation intentions

After commitment to the self-set goals using self-persuasion, the next step was to implement these goals using concrete action plans, that is, implementation intentions (Gollwitzer, 1999). In line with the Theory of Planned Behaviour (Ajzen, 1991; Gollwitzer, 1999), concrete action plans generate stronger intentions that are more likely to translate into observable changes in behaviour (*cf.* the intention-behaviour gap; Sheeran, 2002). Research into the intention-behaviour relationship and goal setting suggests that the more specific our goals, the more effective they are (i.e., larger and more sustainable behavioural changes). For instance, asking people to plan where, when, and how they will make an appointment increased cervical cancer screening (Sheeran and Orbell, 2000). Similarly, reminding people of their higher-order (overarching) goal via implementation intentions has been shown to enhance their self-control in tempting situations for dieting behaviour (Van Koningsbruggen et al., 2011). Further, a meta-analysis into the effects of implementation intentions revealed this technique to have a medium to large effect on goal achievement (Gollwitzer and Sheeran, 2006).

In the intervention applied in the current study, participants identified and wrote down how they would respond in a particular situation in line with their personal goals. Creating an ‘If-Then’ plan for action meant that participants’ responses in a given situation were already intended or planned and that a particular situation at work and/or at home served as a cue for the target response (i.e., less needed to be decided ‘on-the-spot’ or in the situation itself) (as per Sheeran and Orbell, 2000).

##### 2.3.1.3 Self-efficacy

Given that reduced self-efficacy may be an antecedent for burnout (Lemyre et al., 2008; Cherniss, 2017) and that enhanced self-efficacy is associated with improved performance (Bandura, 1990; Bandura and Locke, 2003), fostering participants’ self-efficacy was considered a crucial final step for all goal-setting assignments during the vitality training. At the end of all goal-setting assignments, participants were required to assess how confident they felt about their ability to achieve their developed goal or plan (using a scale of 1–10, from very low to very high). If participants indicated low self-efficacy for a specific goal or plan, they were asked to reflect on which aspect(s) were less achievable and to use insights from their reflection to formulate a goal they felt more capable of achieving. Thus, this self-assessment served two main purposes: (i) to help ensure that participants developed personal goals and plans that were realistic and achievable (and therefore more likely to be acted on) (Locke and Latham, 2002), and (ii) to have participants evaluate their choices and goals at regular intervals during the intervention (i.e., increased self-monitoring). In so doing, participants could realise that they do have the capacity to change aspects of their work-life situation, and, in the context of this intervention, thus have some control over their energy levels (Levin et al., 1998).

#### 2.3.2 Training sessions and method

The intervention consisted of five 2-hour group sessions that were performed in-house, on the organisations’ premises, which were held fortnightly over a 9-week period. The sessions were conducted in small groups of participants within each organisation, with group sizes ranging from seven to 13 participants.^2^ The sessions included the following topics: (1) Personal energy balance analysis; (2) physical and mental energy; (3) working from qualities, values, and goals; (4) personal vitality strategy; and (5) evaluation and maintenance.

During the personal balance analysis, participants were introduced to different types of energy, and examined the activities in their daily life and the impact of these activities on their energy levels. During the session on physical and mental energy, participants were introduced to their mind–body interaction and examined the signals they received from their bodies that might indicate mental and physical fatigue. The third session introduced participants to the idea of ‘flow’ (Csikszentmihalyi and LeFevre, 1989; Nakamura and Csikszentmihalyi, 2014) and the benefits of aligning their daily lives with their qualities, values, and future goals. As such, participants examined their qualities, values, and goals and analysed the fit between these and the activities in their daily life. In the fourth session, participants analysed the various resistances or personal barriers that interfered with achieving their personal goals. In the final session, participants reviewed what they had learned, achieved, and what they would need to remember to maintain the effects of the training and to continue improving their energy balance. For the duration of the training, each participant completed exercises that were contained in a specially-designed workbook, which was structured according to the aforementioned session topics and to the application of the behaviour-change techniques within each session. A description of the scenarios for each session is provided in Appendix 1.

Within each of the training sessions, the intervention method consisted of an initial evaluation of the previous session using a gain frame (Levin et al., 1998), discovering relevant values and goals drawing upon the technique of self-persuasion (as per Müller et al., 2009, 2017), developing personalised plans for change using implementation intentions (as per Sheeran and Orbell, 2000), and ended with participants rating and reviewing their confidence in their ability to achieve their goals set during the session (self-efficacy, as described earlier in the method) (also see the assessment of plan execution self-efficacy described in Scholz et al., 2007). Where confidence was self-identified as low to moderate (versus moderate to high), participants were encouraged to revise their implementation intention, either by making the ‘If’ (situation) or the ‘Then’ (response) components less challenging or by aligning the goal more closely with the value(s) they identified in the self-persuasion exercise.

### 2.4 Trainer

The intervention was delivered by the (independent) developer of the training, who held a PhD in clinical psychology and worked as a researcher, university lecturer, and vitality trainer. Adherence to the intervention method was controlled for, across sessions and participant groups, using a checklist, which was reviewed following each session.

### 2.5 Measures

The Vitality Training Evaluation Scale (VTES) was developed for this study and contained 20 items that measured participants’ subjective experiences of energy, stress, daily life satisfaction, and work capacity, as well as their reported self-efficacy with respect to managing their work-life balance. The primary variables of interest were energy and stress, while secondary variables of interest were daily life satisfaction and work capacity. Self-efficacy with regard to managing one’s work-life balance was included as a possible process measure (i.e., a possible mediator for the effects of the vitality training). Participants responded to all of the VTES items using visual analogue scales ranging from 0 to 100 (never to always). The internal consistencies for each of the factors is described below. A matrix of the correlations between each of the factors at baseline is provided in Table 1.

**Table 1:** Correlation matrix showing bivariate correlations between the vitality training evaluation scale factors at baseline.

	VTES factor			
	2.	3.	4.	5.
*VTES factor*				
1. Energy	−0.35^**^	0.69^***^	0.54^***^	0.22
2. Stress	1	−0.34^**^	−0.38^**^	−0.25^*^
3. Daily life satisfaction		1	0.54^***^	0.26^*^
4. Work capacity			1	0.34^**^
5. Self-efficacy				1

^*^Significant at the 0.05 level. ^**^Significant at the 0.01 level. ^***^Significant at the 0.001 level.

#### 2.5.1 Primary outcomes: energy and stress

Three VTES energy items established participants’ subjective energy levels, which assessed the extent to which participants reported feeling (i) energetic, (ii) physically fit, and (iii) mentally fit. The factor ‘energy’ was a mean of these three items, and had good internal consistency (Cronbach’s alpha: 0.88) (as per Kline, 2000). The mean of the energy items was 57.09 (SD = 22.57), where higher scores reflect higher levels of reported energy.

Two items established the extent to which participants felt stressed and overloaded. Participants responded to these items using visual analogue scales ranging from 0 to 100 (never to always). The factor ‘stress’ was a mean of these two items and was found to have good internal consistency (Cronbach’s alpha: 0.87), the mean being 38.82 (SD = 3.63).

#### 2.5.2 Secondary outcomes: daily life satisfaction and work capacity

Four items measured participants’ subjective daily life satisfaction, including the extent to which they reported (i) being satisfied with their daily life, (ii) paying attention to the activities in their daily life, as well as being (iii) motivated towards and (iv) inspired by the activities in their daily life. A sample item used to assess daily life satisfaction is: “To what extent do you feel motivated for the activities in your daily life?” The factor ‘daily life satisfaction’ was a mean of these four items, which had good internal consistency (Cronbach’s alpha: 0.915) (as per Kline, 2000). The mean of the items for daily life satisfaction was 63.49 (SD = 18.22), where higher scores reflect higher reported daily life satisfaction.

As regards work capacity, six items ascertained the extent to which participants felt (i) motivated, (ii) productive, and (iii) efficient at work, felt (iv) inspired by their work, and the extent to which they felt capable of (v) concentrating and (vi) achieving their goals at work. A sample item used to assess work capacity is: “To what extent do you feel productive at work?” The factor ‘work capacity’ was a mean of these six items, which had good internal consistency (Cronbach’s alpha of 0.89) (as per Kline, 2000). The mean for the work capacity items was 65.56 (SD = 16.86), where higher scores reflect higher reported work capacity.

#### 2.5.3 Process measures: self-efficacy

Participants’ reported ability to manage their work-life balance was ascertained using five items: their perceived (i) ability to change their work-life balance, (ii) influence on having a good work-life balance, (iii) ability to make choices and (iv) set boundaries regarding their work-life balance, and the extent to which (v) their goals regarding their work-life balance were achievable. A sample item used to assess self-efficacy is: “To what extent do you feel capable of setting boundaries with regard to your work-life balance?” Although reliable and valid measures of general self-efficacy already exist (Sherer et al., 1982), self-efficacy is context-specific and a general self-efficacy scale may not be sensitive to changes in a specific domain following training (e.g., see Wang and RiCharde, 1988). Since the vitality training included exercises to help participants manage their personal work-life situation, we chose to develop our own measure of self-efficacy specifically for this study that related to managing one’s work-life balance. The factor ‘self-efficacy’ was the mean of the five aforementioned items and was found to have good internal consistency (Cronbach’s alpha: 0.89) (Kline, 2000). The mean of the self-efficacy items was 67.96 (SD = 18.30), where higher scores reflect higher reported self-efficacy. The item referring to one’s perceived influence on a good work-life balance had the greatest deviation from the factor mean (M = 77.35, SD = 20.87). Although Cronbach’s alpha would increase to 0.91 if this item was deleted, we retained all five items in the factor as the reliability was deemed sufficient, and in order to protect construct validity.

#### 2.5.4 Participant evaluations of the intervention

The perceived effects and value of the vitality training were assessed at the completion of the intervention. Ten items measured the extent to which participants agreed that: (i) the vitality training was helpful; (ii) useful in their everyday life; (iii) had an effect on them; and that (iv) the training effects were lasting. Participants were also asked to rate the extent to which they agreed that they: (v) reached their personal goals during the training; (vi) that the training had an impact on their energy balance; and that (vii) the training would be helpful for other employees in their organisation. Moreover, they were also asked to rate the approach of the training, including the extent to which they agreed that: (viii) the training method was of good quality; and (ix) enjoyable; and that (x) the atmosphere within the group was good. Responses were made using a 5-point Likert scale ranging from 1 (strongly disagree) to 5 (strongly agree), and a sample item is: “The training has had an effect on my energy balance”.

### 2.6 Data cleaning, screening, and analysis strategy

Participants were retained in the analysis to evaluate the intervention if they attended a minimum of three out of the five sessions (*n* = 65 after data cleaning). The retained participants attended a mean of 4.47 sessions, with over 50% of these participants attending all five sessions (52.8%). Of the retained participants, 52 completed all surveys and 51 had complete data for the variables of interest at all three time points (T_0_–T_2_) (*n* = 24 intervention group, *n* = 27 control group). The characteristics of the sample, post data cleaning, were as described in the participants’ section and are summarised for each treatment group in Table 2. Although the mean age and tenure were higher for the control group, these differences were not statistically significant. As a precaution, we also examined the correlations between age and tenure with each of the dependent variables at baseline (T_0_): Significant relationships were not found across the larger part of the dependent variables,^3^ and so age and tenure were not included in our model when examining the effects of the training.

**Table 2:** Mean (SD) and number of participants across demographic variables and reported work experience for each treatment group (*n* = 65).

		Treatment group		
Variable		Control	Intervention	Total
Gender	Male	12	13	25
	Female	25	15	40
Age (years)		48.92 (9.04)	45.79 (8.18)	47.57 (8.76)
Industry	Research	9	5	14
	Commercial	17	16	33
	Semi-government	11	7	18
Tenure (years)		14.99 (10.69)	10.93 (7.71)	13.24 (9.67)
Sessions attended (out of five)		4.41 (0.57)	4.54 (0.65)	4.47 (0.61)

Before conducting the analyses, the data was screened for potential problems, and appeared to meet the assumptions for multivariate analysis of variance (MANOVA). Although no multivariate outliers were detected, one univariate outlier was detected for work capacity at Time Point 2 for a participant in the intervention group (z-score of −3.29). On visual inspection, the value did not appear to be an error, as the low mean for work capacity was consistent with the participant’s scores on the remaining variables, and so the value was retained. At baseline, one participant reported having taken a significant period of sick leave: We considered excluding this participant; however, the overall findings did not change whether the participant was included or excluded from the analysis, and so we decided to retain this participant to maximise statistical power. Although context appears important for the effects of organisational interventions (Randall and Nielsen, 2012), we did not stratify our analyses by the different organisations due to the small sample size.

Given that the VTES factors of energy, stress, daily life satisfaction and work capacity were significantly correlated at baseline (see Table 1), a mixed MANOVA examining the VTES factors was performed to assess changes across time (Time Points 0–2) within and between the treatment groups (control, intervention). Follow-up univariate analysis of variance (ANOVA) and simple effects tests were performed where appropriate. Given that the group sizes were approximately equal and the assumptions were met, we reported Wilks’s lambda, which may be more powerful (see Stevens, 1979). SPSS 25.0 was used for all statistical analyses, and all significance tests were performed using two-tailed tests with alpha set at 0.05. Bonferroni adjustments were not applied for the subsequent ANOVA tests, as this was considered too conservative (i.e., with four dependent variables). However, the simple effects tests applied a Bonferroni correction for multiple comparisons (as described in Zar, 2010).

Because we did not conduct an *a priori* power calculation, and our sample size was reduced following data cleaning, we performed a series of power calculations following our analyses. Power was calculated using G*Power version 3.1, with a significance level (α) of 0.05 and a desired power (1-β) of 0.80. These analyses revealed that for a mixed-MANOVA with two groups and 12 measurements, sample sizes of *N* = 851, *N* = 123, and *N* = 59 would be required to detect small (*f* ^2^ = 0.02), medium (*f* ^2^ = 0.15), and large (*f* ^2^ = 0.35) effect sizes (as per Cohen, 1988), respectfully. In addition, a sensitivity analysis using G*Power revealed that with sample size of *N* = 65, the current study was powered to detect an effect size classified as medium to large (*f* ^2^ = 0.31).

## 3 Results

### 3.1 Intervention effects

**Figure 2:**
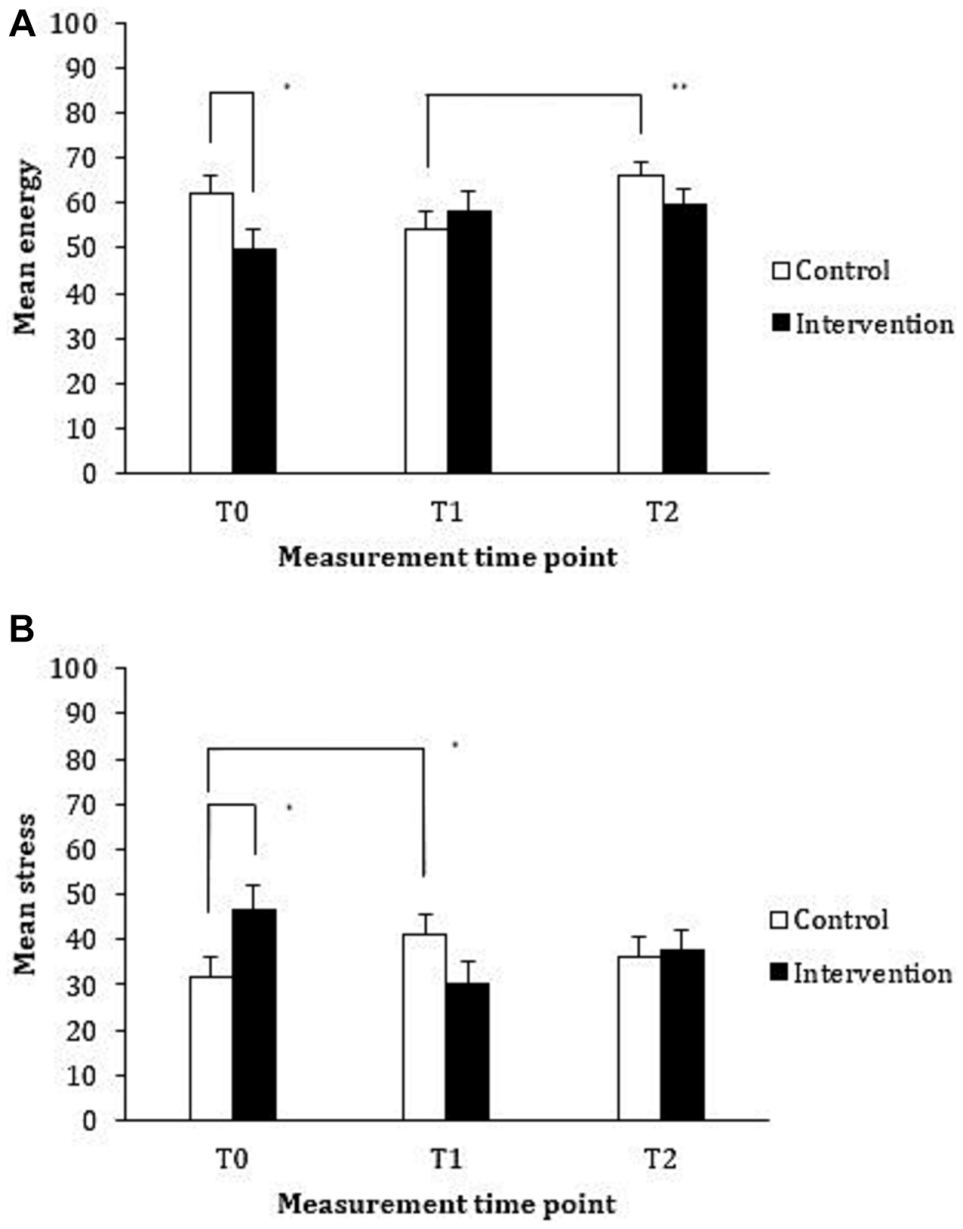
Mean reported energy **(A)** and stress **(B)** levels for the control (waiting list) and intervention (vitality training) groups at baseline (T0), immediately following (T1), and at 3 months following the intervention (T2).

**Figure 3:**
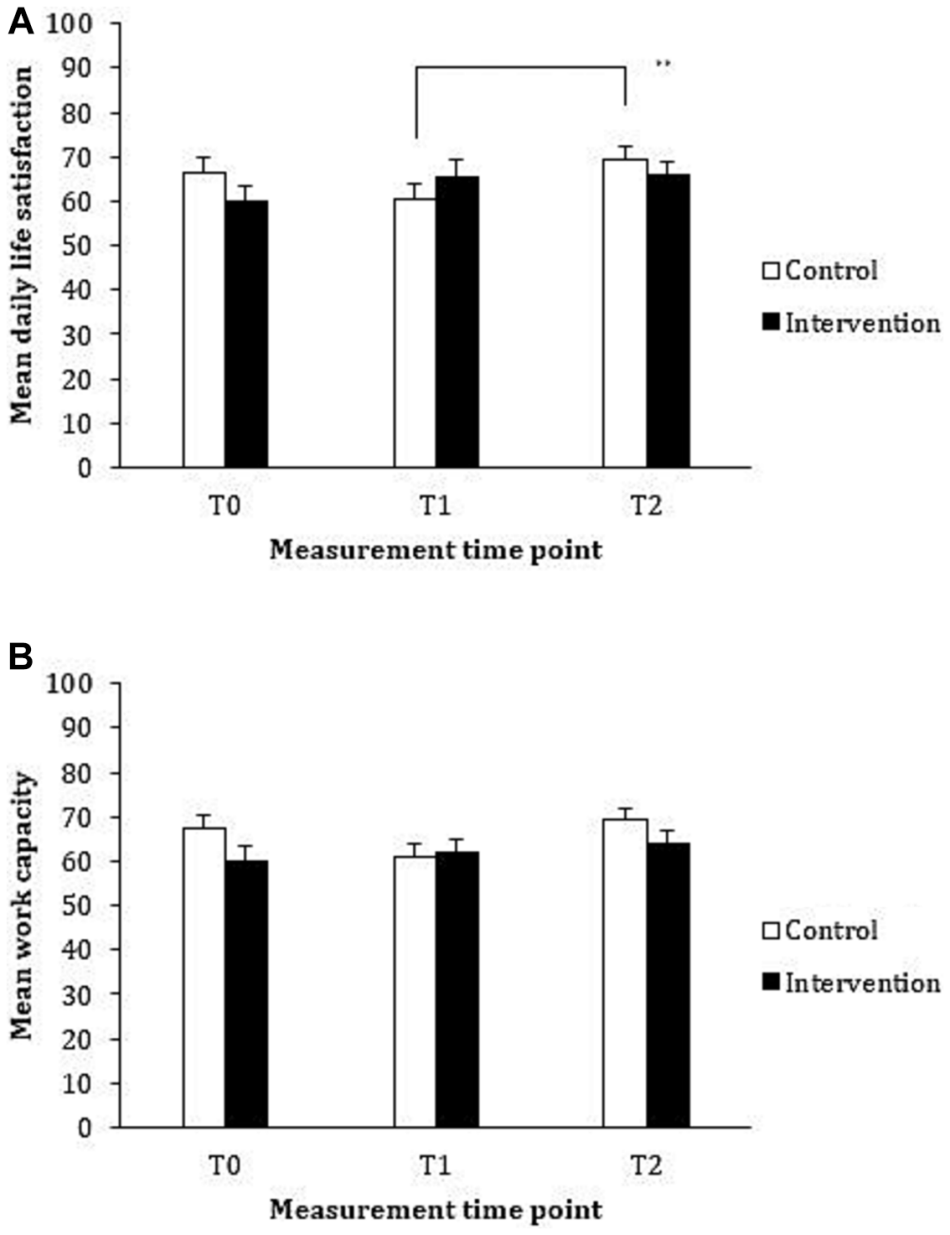
Mean reported daily life satisfaction **(A)** and work capacity **(B)** for the control (waiting list) and intervention (vitality training) groups at baseline (T0), immediately following (T1), and at 3 months following the intervention (T2).

The group means and standard deviations are provided in Table 3; the inter-correlations are presented in Table 4, with all correlations being in the expected directions. The mixed MANOVA assessing changes over time within and between the treatment groups revealed that the main effect for time point was non-significant, Λ = 0.78, *F*(8, 42) = 1.51, *p* = 0.181, *η*_p_^2^ = 0.224; similarly, the main effect for treatment group was also non-significant, Λ = 0.96, *F*(4, 46) = 0.54, *p* = 0.710, *η*_p_^2^ = 0.045. However, the treatment group by time interaction effect was statistically significant, Λ = 0.69, *F*(8, 42) = 2.31, *p* = 0.038, *η*_p_^2^ = 0.306. Given this significant interaction effect for the combination of variables, we subsequently performed univariate ANOVAs for each of the dependent variables of interest using a 2 (treatment group: control, intervention) × 3 (time point: baseline, post-intervention, follow-up) design, with treatment group as the and time point as the within-subjects variable. The between- and within-subject effects for each of the outcome measures are displayed in Table 5 and the interaction effects between treatment group and each time point are displayed in Figures 2, 3.

**Table 3:** Means and standard deviations of outcome variables for each treatment group at each time point.

	Baseline (T0)		Post-training (T1)		Follow-up (T2)	
	Control *n* = 27	Intervention *n* = 24	Control *n* = 27	Intervention *n* = 24	Control *n* = 27	Intervention *n* = 24
*Primary outcome variables*						
Energy	62.01 (18.92)	49.72 (23.52)	54.81 (23.30)	58.11 (19.64)	65.16 (18.13)	59.49 (16.22)
Stress	32.44 (24.11)	46.77 (25.25)	39.93 (27.35)	30.29 (21.39)	37.57 (21.39)	37.50 (24.38)
*Secondary outcome variables*						
Daily life satisfaction	66.48 (16.36)	59.80 (19.56)	60.19 (21.62)	65.46 (14.31)	69.53 (15.79)	65.76 (14.52)
Work capacity	67.25 (15.97)	59.86 (16.66)	62.09 (16.32)	61.69 (16.02)	69.23 (14.17)	64.07 (13.71)

The sample size presented in this table is smaller than what is provided in Figure 1 due to the data cleaning described in section 2.6 and due to the nature of the mixed MANOVA, which applied ‘missing listwise’.

**Table 4:** Matrix of bivariate correlations between treatment group and outcome variables at each time point.

	2.	3.	4.	5.	6.	7.	8.	9.	10.	11.	12.	13.	14.	15.	16.
1. Treatment group^a^	−0.19	0.21	−0.09	−0.23	0.07	0.12	−0.23	0.26^*^	0.03	0.28^*^	−0.19	0.03	−0.13	−0.19	<0.01
2. Energy T0	1	−0.35^**^	0.69^**^	0.54^**^	0.22	0.50^**^	−0.12	0.47^**^	0.46^**^	0.23	0.42^**^	−0.25	0.32^*^	0.26	−0.02
3. Stress T0		1	−0.34^**^	−0.38^**^	−0.25^*^	−0.30^*^	0.31^*^	−0.23	−0.28^*^	−0.12	−0.25	0.36^**^	−0.06	−0.16	−0.07
4. Daily life satisfaction T0			1	0.54^**^	0.26^*^	0.43^**^	−0.22	0.47^**^	0.34^**^	0.31^*^	0.29^*^	−0.31^*^	0.35^*^	0.47^**^	−0.08
5. Work capacity T0				1	0.34^**^	0.41^**^	−0.11	0.40^**^	0.64^**^	0.26^*^	0.41^**^	−0.05	0.35^*^	0.62^**^	0.03
6. Self-efficacy T0					1	0.29^*^	−0.42^**^	0.36^**^	0.29^*^	0.55^**^	0.21	−0.15	0.23	0.36^**^	0.52^**^
7. Energy T1						1	−0.45^**^	0.62^**^	0.68^**^	0.44^**^	0.56^**^	−0.28^*^	0.45^**^	0.35^*^	0.05
8. Stress T1							1	−0.43^**^	−0.45^**^	−0.41^**^	−0.31^*^	0.35^*^	−0.24	−0.23	−0.43^**^
9. Daily life satisfaction T1								1	0.63^**^	0.49^**^	0.49^**^	−0.34^*^	0.63^**^	0.52^**^	0.19
10. Work capacity T1									1	0.41^**^	0.47^**^	−0.10	0.53^**^	0.60^**^	0.19
11. Self-efficacy T1										1	0.40^**^	−0.38^**^	0.44^**^	0.43^**^	0.59^**^
12. Energy T2											1	−0.44^**^	0.63^**^	0.59^**^	0.30^*^
13. Stress T2												1	−0.44^**^	−0.30^*^	−0.34^*^
14. Daily life satisfaction T2													1	0.77^**^	0.38^**^
15. Work capacity T2														1	0.27
16. Self-efficacy T2															1

^a^A positive correlation with treatment group reflects a higher score on the outcome variable of interest for the intervention group. ^*^Significant at the 0.05 level. ^**^Significant at the 0.01 level.

**Table 5:** Between- and within-subject effects for univariate ANOVAs examining the effect of the vitality training.

Measure		Effect	
*Primary outcome variables*			
	Energy	Treatment group	*F*(1, 50) = 1.25, *p* = 0.269, *η*_p_^2^ = 0.024
		Time point	*F*(2, 100) = 3.47, *p* = 0.035, *η*_p_^2^ = 0.065
		Treatment group × time point	*F*(2, 100) = 4.16, *p* = 0.018, *η*_p_^2^ = 0.077
	Stress	Treatment group	*F*(1, 50) = 0.14, *p* = 0.712, *η*_p_^2^ = 0.003
		Time point	*F*(2, 100) = 0.46, *p* = 0.633, *η*_p_^2^ = 0.009
		Treatment group × time point	*F*(2, 100) = 5.76, *p* = 0.004, *η*_p_^2^ = 0.103
*Secondary outcome variables*			
	Daily life satisfaction	Treatment group	*F*(1, 49) = 0.19, *p* = 0.664, *η*_p_^2^ = 0.004
		Time point	*F*(2, 98) = 2.46, *p* = 0.091, *η*_p_^2^ = 0.048
		Treatment group × time point	*F*(2, 98) = 3.28, *p* = 0.042, *η*_p_^2^ = 0.063
	Work capacity	Treatment group	*F*(1, 50) = 1.16, *p* = 0.287, *η*_p_^2^ = 0.023
		Time point	*F*(2, 100) = 4.35, *p* = 0.015, *η*_p_^2^ = 0.080
		Treatment group × time point	*F*(2, 100) = 2.77, *p* = 0.067, *η*_p_^2^ = 0.052

#### 3.1.1 Energy

An examination of the impact of the training on reported energy levels revealed a significant main effect for time point. Pairwise comparisons revealed that reported energy was significantly higher at Time Point 2 than at Time Point 1. However, the main effect can be better understood when examining the significant interaction effect between treatment group and time point, displayed in Figure 2A. Pairwise comparisons revealed that the intervention group reported significantly lower levels of energy than the control group prior to the intervention (*p* = 0.036, 95% CI [−24.32, −0.83]); however, there was no significant difference between treatment groups immediately following the intervention (*p* = 0.510, 95% CI [−8.08, 16.07]) or at the 3-month follow-up (*p* = 0.190, 95% CI [−16.07, 3.28]). Pairwise comparisons examining the differences between time point for each group revealed that although the intervention group reported higher levels of energy following the intervention when compared to baseline, this improvement in reported energy did not reach statistical significance (*p* = 0.235, 95% CI [−3.18, 19.96]). For the control group, a significant improvement in reported energy levels was observed following exposure to the intervention (i.e., when comparing energy at Time Points 1 and 2), (*p* = 0.003, 95% CI [3.34, 20.18]). Thus, it appears that the significant main effect for time point is largely driven by the significant improvement in reported energy by the control group following training. No other differences were statistically significant.

#### 3.1.2 Stress

The impact of the training on reported stress levels revealed a significant interaction between treatment group and time point (see Figure 2B; all within and between-group effects are reported in Table 5). Pairwise comparisons revealed that the intervention group reported significantly higher levels of stress than the control group prior to the intervention (*p* = 0.032, 95% CI [1.39, 28.90]); however, the differences between treatment groups immediately following the intervention (*p* = 0.130, 95% CI [−24.46, 3.22]) and at follow-up (*p* = 0.857, 95% CI [−11.75, 14.08]) were not statistically significant. Pairwise comparisons between time points for each of the treatment groups revealed that there was a significant improvement in reported stress levels immediately following the training for the intervention group (i.e., from Time Point 0 to Time Point 1) (*p* = 0.017, 95% CI [−30.64, −2.32]). Although participants in the control group reported lower levels of stress following the training, this improvement in reported stress levels was not statistically significant (*p* > 0.99, 95% CI [−17.24, 8.10]). No other differences were statistically significant.

#### 3.1.3 Daily life satisfaction

Examining the effect of the training on reported daily life satisfaction revealed no main effects; however, a significant treatment group by time point interaction emerged (as displayed in Figure 3A). Although the differences between treatment groups at each time point during the trial were in the expected direction, pairwise comparisons revealed that these differences did not reach statistical significance. When examining the differences across time point for each of the treatment groups, the improvement in reported daily life satisfaction for the intervention group from pre- to post- training was not statistically significant (*p* = 0.394, 95% CI [−3.48, 14.79]). However, the improvement in reported daily life satisfaction for the control group following training (i.e., from Time Point 1 to Time Point 2) was statistically significant (*p* = 0.004, 95% CI [2.52, 16.14]). No other differences were statistically significant.

#### 3.1.4 Work capacity

Examining the impact of the training on reported work capacity revealed no main effect for treatment group, but a main effect for time point was revealed. Pairwise comparisons revealed that work capacity significantly improved from Time Point 1 to Time Point 2 (*p* = 0.018, 95% CI [0.76, 10.15]). Although the interaction effect between treatment group and time point was in the predicted direction (displayed in Figure 3B), the interaction effect did not reach statistical significance.

### 3.2 Self-efficacy as a mechanism for intervention effects

To explore whether reported self-efficacy mediated the effect of the vitality training on our primary outcome measures of energy and stress, a bootstrapping approach (Preacher and Hayes, 2004) was performed using the PROCESS macro in SPSS. The conducted bootstrapping technique involved repeatedly sampling (10,000 times) on the dataset to estimate the indirect effect, and using bias-corrected confidence intervals. The (bias-corrected) 95% CIs were then examined to see whether they contained zero (i.e., where a confidence interval does not contain zero, this indicates statistical significance).

We examined whether increases in self-efficacy regarding managing one’s work-life balance mediated increases in reported energy levels in the training group. Specifically, we examined the extent to which increases in self-efficacy from baseline to post-intervention mediated the increase in energy observed in the intervention group. To model changes in energy, baseline scores were entered into the bootstrap analysis as a covariate, and post-intervention scores were entered as the dependent variable; similarly, to model changes in self-efficacy, baseline scores were entered as a covariate, and post-intervention scores were entered as a mediator. Treatment group (control, intervention) was entered as the independent variable. The indirect effect of treatment group on reported energy levels following the training through self-efficacy was significant (see Table 6).

**Table 6:** Bootstrap analyses for detecting the indirect effect of the vitality training on primary outcome variables.

		Bootstrap estimate		BC 95% CI^a^	
Outcome variable	Mediator variable	Estimate	SE	Lower	Upper
Energy^b^ T0–T1	Self-efficacy T0–T1	3.49	2.38	0.18	9.33
Stress^c^ T0–T1	Self-efficacy T0–T1	−2.26	2.10	−6.88	1.56

^a^BC = Bias-corrected confidence intervals (i.e., corrected for the median). Confidence intervals containing zero represent non-significant effects at the 0.05 level of significance; 10,000 bootstrap samples. ^b^Positive estimates for energy reflect that increases in energy are predicted for the intervention group. ^c^Negative estimates for stress reflect that reductions in stress are predicted for the intervention group.

A similar procedure was performed for reported stress levels, and the results of these analyses are displayed in Table 6. The indirect effect of treatment group on reported stress levels following training through self-efficacy was not statistically significant, suggesting that self-efficacy did not mediate the effects of the training on reported stress.

### 3.3 Participant evaluations

A self-reported evaluation of the intervention by the participants (using a 5-point Likert scale) revealed that they believed that the vitality training was helpful (M = 3.96, SD = 0.52), useful in their everyday life (M = 3.91, SD = 0.49), had an effect on them (M = 4.04, SD = 0.44), and had lasting effects (M = 3.45, SD = 0.61). In particular, participants moderately agreed that they reached their personal goals made during the training (M = 3.28, SD = 0.60) and that the vitality training had an impact on their energy levels (M = 3.55, SD = 0.64). In terms of the approach of the training, participants reported that the method was of good quality (M = 4.06, SD = 0.56), and enjoyable (M = 3.81, SD = 0.65), and that the atmosphere within the group was good (M = 4.38, SD = 0.56). Finally, participants thought that the vitality training would be helpful for other employees in their organisation (M = 3.92, SD = 0.62).

## 4 Discussion

The current research sought to explore the effects of a 9-week intervention in the form of a vitality training employing behaviour-change techniques on reported levels of energy, stress, daily life satisfaction, and work capacity using an RCT. In addition, we sought to examine whether any immediate effects of the intervention were maintained 3 months later for those in the intervention group, and whether the effects of the intervention were replicated in the control group over this time period. Finally, we were interested in exploring whether self-efficacy to manage one’s work-life balance mediated the effects of the intervention on reported energy and stress, and explored the acceptability of the training as reported by participants.

### 4.1 The effects of the intervention

A key finding was the significant interaction effect between treatment group and measurement time point for the combination of reported energy, stress, daily life satisfaction, and work capacity, thereby supporting *Hypothesis 1*. Follow-up analyses revealed significant interaction effects between treatment group and measurement time point for reported energy, stress, and daily life satisfaction, but not for work capacity. Reported energy levels increased for both groups after they, respectively, completed the vitality training; however, only the observed increase in reported energy for the control group reached statistical significance, herewith providing partial support for *Hypothesis 2*a. Similarly, reported stress levels improved for both groups following completion of the training; however, only the reduction in reported stress levels for the intervention group reached statistical significance, thereby providing partial support for *Hypothesis 2*b. Partial support was found for *Hypothesis 3*a regarding the secondary outcome measure of daily life satisfaction: Reported daily life satisfaction increased for both groups after, respectively, completing the vitality training; however, only the increase for the control group reached statistical significance. Although the differences in reported work capacity within groups across time were in the predicted direction, the interaction did not reach statistical significance, and thus *Hypothesis 3*b was not supported by our data.

Taken together, the findings of the current evaluation suggest that a vitality training grounded in evidence-based behaviour-change techniques shows some promise as an approach for improving indicators associated with burnout, as measured at the completion of the vitality training. It is also important to note that significant decrements were not observed in the intervention group at 3 months following their completion of the training, suggesting that the effects of the training were sustained over this time period. This evidence is encouraging for the potential benefits of a vitality training employing the behaviour-change techniques of self-persuasion (see Aronson, 1999; Müller et al., 2009, 2017), implementation intentions (see Gollwitzer, 1999; Sheeran and Orbell, 2000; Van Koningsbruggen et al., 2011), and self-efficacy to target indicators associated with burnout. Although preliminary, this evidence is particularly encouraging since the vitality training was a relatively short intervention and the participants did not score extremely high on reported symptomology prior to intervention; that is, greater changes or effects might have been observed immediately following training, had participants reported symptoms that were more severe prior to the intervention (*cf.* Maricuţoiu et al., 2016). The implications of these findings are valuable from a workplace wellbeing and sustainable career (De Vos et al., 2020; Van der Heijden et al., 2020) perspective, given the reported incidence and prevalence of work-related stress and burnout and its association with increased mental and physical symptoms for individuals (Nixon et al., 2011; Van Zwieten et al., 2014) and increased absenteeism and commitment for organisations (Golembiewski et al., 1998).

It is interesting that the improvements observed following training did not reach statistical significance for both groups across all of the variables. It is plausible that the lack of consistent effects observed across treatment groups are attributable to insufficient statistical power, but could also be explained by the differences observed between these groups prior to intervention. For instance, at baseline, the overall pattern of scores across the measures suggested that participants in the intervention group had worse symptoms overall than participants in the control group (i.e., lower levels of energy, daily life satisfaction, and work capacity, and higher levels of stress), and the significant improvement observed for this group following intervention was for the outcome of stress. On the other hand, for the control group – whose overall pattern of scores across the measures was more favourable – significant improvements were observed for reported levels of energy and daily life satisfaction. It could be that baseline stress levels moderate the effects of the training, whereby the effect of the training on stress is stronger for those with higher baseline stress levels, while the effect on energy is stronger for those with lower baseline stress levels. Accordingly, it could be that participants prioritise and tailor the focus of the training to these baseline needs. The possible moderating effects of baseline energy and stress could be empirically tested, and it is recommended that subsequent evaluations also record and explore the role of a participant’s focus during the training on its effects.

In addition, the finding that training did not have a significant effect on participants’ reported work capacity could be explained by insufficient power; however, this could also be explained by the relatively short intervention and assessment periods. That is, it may take substantially longer to see significant improvements in the aspects of work capacity assessed – particularly for concentration, productivity, and effectiveness, which are indicators of work performance. That we obtained preliminary evidence for improvements to reported energy following training may mean that flow-on effects could be observed for work capacity over a longer time period, and this should be examined in a subsequent evaluation.

### 4.2 The role of self-efficacy

Exploring the indirect effect of the intervention on reported energy and stress provided preliminary support for a mediating role of self-efficacy in the effects of the intervention on reported energy; however, this effect was not found for reported stress. That self-efficacy was found to mediate the relationship between the effects of the intervention and reported energy provides preliminary evidence for the value of interventions targeting self-efficacy regarding managing one’s work-life balance to increase energy levels. However, since self-efficacy was not directly manipulated and was measured at the same time point as energy in this study, it is recommended that future work in this area establishes the causal ordering of the effect – particularly as increasing evidence is emerging for reciprocal relationships involving self-efficacy (Simbula et al., 2011; Granziera and Perera, 2019).

That self-efficacy was not found to mediate the effect of the training on stress may be an artefact of the measurements used (e.g., if stress evokes greater affective evaluations, rather than cognitive evaluations), or it may be that the reported effects are underestimated since other factors could influence stress. The explanation that we offer, however, is that the vitality training topics and activities did not focus on stress directly – rather, the focus was on adaptive responses that could increase energy (i.e., topics included: energy balance analysis; physical and mental energy). Thus, while the vitality training may have a positive effect on stress, it is plausible that this does not occur via enhanced self-efficacy about managing one’s work-life balance.

### 4.3 Participants’ evaluation of the training

Participants evaluated the vitality training favourably, with the average ratings suggesting that they liked the training method and the atmosphere, and that they saw value in the training for themselves and other employees in their organisation. Importantly, on average, they agreed that the training had had a positive and lasting effect on them, and that their energy balance was improved. What is less clear from the quantitative ratings, is what specific improvements to the intervention participants would recommend in order to strengthen their experience and the perceived effectiveness of the training. Overall, however, the vitality training appears to be an acceptable intervention from the perspective of participants.

### 4.4 Strengths and limitations of the current research

The vitality training evaluated in the current research has many strengths, including its scientific basis, and its relatively short duration and ease of implementation. Importantly, the intervention may have moderate effects on reported energy, stress, and daily life satisfaction, with these effects maintained 3 months after the intervention. Regarding the methodology of the current research, the main strengths are in the design (using an RCT), and the congruence between the targets of the intervention and the outcome measures of interest. Previous meta-analyses of the effects of burnout interventions have acknowledged a lack of control conditions and random allocation of participants to treatment groups, herewith limiting the validity and reliability of the findings of such evaluations (*cf.* Maricuţoiu et al., 2016). As such, the use of an RCT in the current research makes a significant contribution to the literature in this area. Similarly, experts in this field have called for more tailored interventions, which consider the diverse range of experiences and problems that individuals may experience when confronted with burnout symptoms. Drawing upon the behaviour-change techniques of self-persuasion (see Aronson, 1999; Müller et al., 2009, 2017) and implementation intentions (see Gollwitzer, 1999; Sheeran and Orbell, 2000; Van Koningsbruggen et al., 2011) – where participants self-generated their reasons and strategies for change – ensured an evidence-based approach, while providing sufficient flexibility for participants to tailor the intervention to their personal work-life situation. This approach, combined with the recruitment of participants across three distinct organisations, likely increases the external validity of the findings with respect to other work-life situations. Thus, the current intervention makes a significant contribution by targeting behaviour in the form of establishing adaptive responses to the work-life situation – rather than targeting coping strategies, which have been criticised previously (*cf.* Le Blanc and Schaufeli, 2008) – and by allowing a more tailored approach to changing the precursors to burnout. The study is novel and makes a valuable contribution to an important area of vitality in the workplace, which may be particularly important at this time given the significant disruptions to workloads following the covid-19 pandemic (Kranenburg et al., 2022; Collie, 2023).

Despite the aforementioned strengths, there are also limitations to the current research that must be acknowledged. In interpreting our findings, it is important to recognise potential limitations associated with the employed measures. The outcome measures lack formal validation, and while their internal consistencies and interrelationships suggest meaningful associations, future research with a larger sample size should undertake a comprehensive examination of the underlying factor structure to establish a more robust foundation for interpretation. An apparent limitation is the relatively small number of participants who were recruited and retained, and who completed all measures throughout the study, which increases the chance of failing to detect an effect of the training where there is one. Given that statistically significant differences were detected in the current study, it appears there was sufficient statistical power to explore the impact of the training. However, since there was substantial variability in the data and the study was underpowered to detect medium to small effect sizes, it is recommended that a larger-scale trial be conducted to (i) confirm the impacts reported, (ii) potentially allow medium effects to be detected, and (iii) to enable sub-group analyses. In addition, although participants of this study were recruited from diverse types of organisations – namely, government, education, and commercial organisations – these do not represent all types of organisations, which means that our findings may not generalise to other settings. Similarly, participants self-selected to participate in the training and the research. While this is fairly common practice (e.g., for randomised clinical trials Martínez-Mesa et al., 2016) and generally considered ethical as it reflects the voluntary nature of participation, this could have introduced selection bias and may imply that the findings cannot be generalised to other groups. Furthermore, the participants did not report high symptomology prior to the intervention. This has been noted elsewhere as a limitation of burnout intervention evaluations more broadly (*cf.* Maricuţoiu et al., 2016), and in our case could have led to an under-assessment of the real effect of the vitality training.

Another aspect of the research that limits its internal validity is that participants developed and worked on different personal goals during the intervention. Although this was the objective of the current intervention (i.e., to allow individual tailoring), this makes it difficult to make any conclusions about the specific outcomes or target behaviours that contributed to the effectiveness of the intervention. Similarly, as several behaviour-change techniques were implemented in the vitality training, it is hard to isolate which specific technique(s) contributed to the observed effects of the intervention, lowering the internal validity of the current research and restricting the suggestions that can be made about which elements should be harnessed in future interventions. Finally, while a waitlist control group was employed in the current research, no alternative active control or intervention group was included, herewith limiting the internal validity of the study. This makes it difficult to establish whether just participating in any intervention was superior to being on a waitlist control group (e.g., see quantifications of the Hawthorne effect using placebo-controlled trials; McCarney et al., 2007), rather than establishing that the behaviour-change elements – in particular – were effective. In addition, it would be useful to examine changes to the outcome measures at an even greater latency following the intervention: If it is the case that employees learn how to make changes over time, it is plausible that greater improvements to energy levels may be seen at a later stage. Another limitation of the current research is that observable behaviour was not measured. The reliance on self-reported measures only, instead of including observable behaviour, can be seen as a limitation that has been acknowledged previously (e.g., see a review of the intention-behaviour gap, Sheeran, 2002). On the other hand, as work-related stress tends to be conceptualised as an individual’s experience of the work situation (e.g., see Maslach et al., 2009), it could be argued that the omission of objective measures may not be hugely limiting in this case. However, future extensions of this work could include gathering objective data on the behaviour(s) that participants select to work on during the vitality training, as well as objective measures of productivity and absenteeism.

### 4.5 Practical implications

JD-R theory, being the underlying framework of our empirical study, can also be used to guide the practical implications of our study. Overall, interventions aimed at increasing employee wellbeing, and through this, enhance job and organisational performance, may often take place at an organisational level, for example, by improving the balance between job demands and job resources. However, our study has indicated that a focus on individual-level interventions, such as the 9-week vitality training proposed in our study, can pay off as well. In particular, this example of proactive vitality management, that employs well-known behaviour-change techniques of self-persuasion, implementation intentions, and self-efficacy, is a fruitful human resource management (HRM) practice for enhancing desired employee outcomes (i.e., increased energy and reduced stress, being primary outcomes in our study, and increased daily life satisfaction, being a secondary outcome in our study). Moreover, building on our findings, we invite practitioners in the field of (sustainable) HRM, and particularly those intending to enhance employee vitality, to take account of employees’ self-efficacy to manage their work-life balance as this factor plays an important role in translating the primary effects of the intervention into the desired outcome of increased energy. Obviously, the implementation of proactive vitality management stands or falls with a supportive line manager who helps the employee with tailor-made work-life balance strategies. At the same time, employees themselves need to carry responsibility for protecting their work-life balance, for instance by separating work and family time, duties, and activities, or by exploring opportunities to enrich each other. This dual responsibility, wherein both employer and employee objectives are aligned, is needed to foster sustainable careers (De Vos et al., 2020) wherein both health and happiness (employee-related indicators of sustainable careers; Van der Heijden, 2005), and productivity (employer-related indicator of sustainable careers; *ibid.*) are all prioritised.

## 5 Conclusion

The present study extended previous investigations into interventions for vitality by exploring the effects of a vitality training that employed behaviour-change techniques using an RCT. The results of the current research provide preliminary evidence for the benefits of employing the behaviour-change techniques of self-persuasion, implementation intentions, and self-efficacy in a vitality training for reported energy, stress, and daily life satisfaction levels, without significant decrements to these indicators 3 months after the completion of training. However, the effects of the training on work capacity were less clear and may need to be assessed over longer time periods with a larger sample. The current evaluation identified self-efficacy to manage one’s work-life balance as playing a possible mediating role in the effects of the intervention on reported energy; however, an indirect effect of the training through self-efficacy was not observed for changes to reported stress. Future extensions of this work should focus on examining the relative role that each of the behaviour-change techniques and training elements play in producing these effects, and in testing the causal ordering of the role of self-efficacy. Such research could make significant contributions to developing much needed effective interventions to enhancing vitality and addressing symptoms associated with burnout.

## Data availability statement

The raw data supporting the conclusions of this article will be made available by the authors, without undue reservation.

## Ethics statement

The study was conducted in the Netherlands in full compliance with the applicable rules of the institutional review board (Ethics Committee Faculty of Social Sciences, Radboud University, the Netherlands) and informed consent was obtained from all participants. All ethical codes as maintained in the NIP (the Dutch Association of Psychologists), the American Psychological Association, and the British Psychological Society were followed.

## Author contributions

BP: Conceptualization, Data curation, Formal analysis, Visualization, Writing – original draft, Writing – review & editing. ML: Conceptualization, Investigation, Methodology, Writing – original draft, Writing – review & editing. PP: Conceptualization, Investigation, Methodology, Writing – original draft, Writing – review & editing. BH: Conceptualization, Investigation, Methodology, Writing – original draft, Writing – review & editing.

## Footnotes

^1^ANOVA, analysis of variance; BC, bias corrected; CI, confidence interval; CBT, cognitive behaviour therapy; MBI, Maslach Burnout Inventory; MANOVA, multivariate analysis of variance; RCT, randomised controlled trial; SF-36, RAND 36-Item Health Survey; SVS, Subjective Vitality Scale; T0, Time Point 0 (i.e., baseline data collection period); T1, Time Point 1 (i.e., post-intervention data collection period); T2, Time Point 2 (i.e., follow-up data collection period); UWES, Utrecht Work Engagement Scale; VTES, Vitality Training Evaluation Scale.

^2^Specifically: the educational organisation included one intervention group (*n* = 10) and one waitlist control group (*n* = 11); the commercial organisation included two intervention groups (*n* = 21) and two waitlist control groups (*n* = 22); and the government organisation included one intervention (*n* = 7) and one waitlist control group (*n* = 13).

^3^In particular, age was positively related to daily life satisfaction (*r* = 0.28, *p* = 0.026). Regarding tenure: a significant negative relationship was observed with stress (*r* = −0.30, *p* = 0.014) and a significant positive relationship was observed with work capacity (*r* = 0.31, *p* = 0.013). All other correlations were non-significant.
