# Supplementary material for: Train to sustain: a randomised controlled trial evaluation of a vitality training employing behaviour-change techniques
Source: Front Psychol. 2024 Jan 16;14:1320826. doi: 10.3389/fpsyg.2023.1320826 (PMC10826839; doi:10.3389/fpsyg.2023.1320826)
Supplement: Supplementary file 1 [file Data_Sheet_1.docx]

# Appendix 1: Training scenario descriptions

## Personal energy balance analysis

During this session, participants examined their current energy levels across physical, mental, emotional, and life, and also rated how much energy they had for their work and for the activities in their daily life. Following this, participants generated a list of activities in their daily life, and then mapped these onto an energy balance scale according to whether they considered the activity to contribute positively or negatively to their energy levels. Participants then reflected on broad changes they would like to make before making these changes more specific.

These activities formed the basis for participants to develop their personalised plan for change, using the behaviour change techniques of self-persuasion, implementation intentions, and self-efficacy (described in section 2.3.1 Techniques central to the training method).

## Physical and mental energy

The start of the session included an individual reflection and evaluation on progress since the previous session, primarily using a gain frame.

During this session, participants were introduced to personal needs and their mind-body interaction. This included examining what their current needs might be to manage their energy levels, what signals they receive from their bodies that might indicate mental and physical fatigue, and how they react during times of reduced energy or increased stress. Participants then reflected on positive responses or changes that they could make in response to these signals.

These activities formed the basis for participants to develop their personalised plan for change, using the behaviour change techniques of self-persuasion, implementation intentions, and self-efficacy (described in section 2.3.1 Techniques central to the training method).

## Working from qualities, values, and goals

The start of the session included an individual reflection and evaluation on progress since the previous session, primarily using a gain frame.

During this session, participants reflected on their qualities and how these translate in activities in their work and daily lives, including reflecting on potential pitfalls of their identified qualities. Similarly, participants reflected on their values and how these translate in activities in their work and daily lives. Next, participants reflected on and noted their future goals, and examined what activities or steps could be taken at the current time to work towards these goals. The final activity required participants to examine current activities that do not align with their qualities, values and goals, and to consider how these could be changed.

These activities formed the basis for participants to develop their personalised plan for change, using the behaviour change techniques of self-persuasion, implementation intentions, and self-efficacy (described in section 2.3.1 Techniques central to the training method).

## Personal vitality strategy

The start of the session included an individual reflection and evaluation on progress since the previous session, primarily using a gain frame.

During this session, participants reflected on what has already changed in their daily life and what appears to be difficult to change. Participants also explored and identified what they believe they need to do in order to achieve lasting vitality and what actions would lead to them not achieving this. They then examined and noted various personal barriers that they felt interfered with them achieving their personal goals (e.g., moods and situations).

These activities formed the basis for participants to develop their personalised plan for change, using the behaviour change techniques of self-persuasion, implementation intentions, and self-efficacy (described in section 2.3.1 Techniques central to the training method).

## Evaluation and maintenance

During this session, participants reflected on the goals they had set themselves, the effort they had put into achieving their goals, and their achievements and insights gained during the course of the training. Participants also revisited their current needs and areas of ongoing focus. Finally, participants brought these reflections together to note what they want to continue doing to maintain any positive effects and areas they would like to continue working on going forward.
